# Pitaya Nutrition, Biology, and Biotechnology: A Review

**DOI:** 10.3390/ijms241813986

**Published:** 2023-09-12

**Authors:** Kamran Shah, Jiayi Chen, Jiaxuan Chen, Yonghua Qin

**Affiliations:** 1 Guangdong Provincial Key Laboratory of Postharvest Science of Fruits and Vegetables, Ministry of Agriculture and Rural Affairs, College of Horticulture, South China Agricultural University, Guangzhou 510642, China; kamranshah801@scau.edu.cn (K.S.); chenjiayi98@stu.scau.edu.cn (J.C.); jxchen0127@163.com (J.C.); 2 Key Laboratory of Biology and Genetic Improvement of Horticultural Crops, Ministry of Agriculture and Rural Affairs, College of Horticulture, South China Agricultural University, Guangzhou 510642, China

**Keywords:** biotechnology, botany, Cactaceae, *Hylocereus*, micropropagation, pitaya, taxonomy

## Abstract

Pitaya (*Hylocereus* spp.) is a member of the cactus family that is native to Central and South America but is now cultivated throughout the sub-tropical and tropical regions of the world. It is of great importance due to its nutritional, ornamental, coloring, medicinal, industrial, and high consumption values. In order to effectively utilize and develop the available genetic resources, it is necessary to appreciate and understand studies pertaining to the usage, origin, nutrition, diversity, evaluation, characterization, conservation, taxonomy, and systematics of the genus *Hylocereus*. Additionally, to gain a basic understanding of the biology of the plant, this review has also discussed how biotechnological tools, such as cell and tissue culture, micropropagation (i.e., somatic embryogenesis, organogenesis, somaclonal variation, mutagenesis, androgenesis, gynogenesis, and altered ploidy), virus-induced gene silencing, and molecular marker technology, have been used to enhance pitaya germplasm.

## 1. Introduction, Origin, History, and Domestication

Pitaya is a tropical, climbing, and perennial fruit crop belonging to the genus *Hylocereus* or *Seleniereus* (Cactaceae) under the Caryophyllales order. The former genus of pitaya, *Hylocereus*, originates from the Greek word “hyle”, which means “woody”, and the Latin word “cereus”, which means “waxen”, while the new genus *Selenicereus* is derived from the Greek “Selene”, which refers to nocturnal flowers. The name of the species ‘*undatus*’ means “wavy edges”, which is comparable to the stem’s rib-like structure [1]. Pitaya is also known as pitaya roja in Central America, pitahaya in Mexico, and dragon fruit in Vietnam due to the leather-like texture of the fruit peel [2]. In the previous literature, “pitaya” is a Haitian word meaning scaly fruit [3]. In Mexico, “pitahaya” refers to epiphytic cacti like *Hylocereus*, whereas “pitaya” refers to fruits of column-like cacti, while in South and Central America, “pitaya” and “pitahaya” have similar meanings [3].

After the discovery of America, family members of the Cactaceae were recognized in Europe. Fernández de Oviedo, Gonzalo, the first New World narrator, included a description of pitahaya in his 1535 book *General and Natural History of the Indies* [4], and the described fruit was referred to as pitaya fruit in Mexico [5,6]. *Hylocereus* was mentioned in Britton’s *Flora of Bermuda* in 1918 and was considered native to tropical America and Mexico before being introduced to Florida and the West Indies [7]. Pitaya originated in areas of Mexico and Central America with rainfall ranging from 350 to 2000 mm and a few meters to 1840 m above sea level [3]. Pitaya was introduced to Taiwan 300 years ago and Vietnam in the 19th century, but the industry in Taiwan started after the introduction of new self-compatible varieties from Vietnam in 1983 [8]. Pitaya was introduced to Indochina in 1860, Southeast Asian countries in the 16th century [9], and Israel in 1984 [10,11,12]. Since then, they have conducted several genetic and physiological studies to increase production and fruit quality characteristics [13,14,15,16,17].

Pitaya is recently domesticated, and there are three main cultivars, *H. undatus* (white pulp with red peel), *H. polyrhizus*/*monacanthus*/*H. costaricensis* (red pulp with red peel), and *H. megalanthus* (white pulp with yellow peel), which are cultivated on a large scale in many regions of the world [13,18]. *H. undatus* is native to Columbia, Mexico, and South America [5,19,20]; *H. megalanthus* is native to Bolivia, Peru, Ecuador, Colombia, and Venezuela [21]; and *H. polyrhizus* is native to Mexico [19,22].

Research on pitaya is gradually increasing, with a search on sciencedirect.com revealing 95 studies in 2019, 112 in 2020, 178 in 2021, 225 in 2022, and already 186 in 2023. This makes the release of the current review important and timely.

## 2. Nutritional Composition

*Hylocereus* has various species and cultivars cultivated throughout the sub-tropical and tropical regions of the world, depending on the nutritional values, acclimatization, compatibility, and demands of the people. The nutritional composition between different species, geographical locations, and methods of determination may differ. Pitaya is rich in multiple natural compounds, such as betalains, polyphenols, phenolic acid, flavonoids, fatty acids, terpenes, and sterols. It is also rich in vitamins such as B1, B2, B3, and C; minerals such as potassium, sodium, calcium, phosphorus, and iron; protein; fat; carbohydrates; fiber; phytoalbumin; and carotenes [23]. The nutritional compositions of different species of pitaya are presented in Table 1. Briefly, each 100.0 g of *H. undatus* fruit pulp contains 28.65 mg GAE total phenolic content [24], 6.26 g carbohydrates, 0.83 dietary fiber, 6.06 g total sugar, 0.94 g protein, 5.64 mg vitamin C, 0.57 g fat, 32.7 mg sorbitol, 45.7 mg calcium, 29.9 mg phosphorus, 45.9 mg magnesium, 193.0 mg potassium, 0.87 mg iron, 4.50 mg sodium, 0.34 mg zinc, 2.15 g fructose, 0.43 mg niacin, and 100.0 µg vitamin E [25,26]. Each 100.0 g *H. polyrhizus* fruit pulp contains 24.22 mg GAE total phenolic content [24], 5.60 g total sugar, 5.97 g carbohydrate, 0.89 g protein, 1.01 g dietary fiber, betacyanin 28.0 mg, 0.78 mg iron, 29.2 mg phosphorous, 33.2 mg magnesium, 158.29 mg potassium, 14.30 mg sodium, 0.29 mg zinc, 31.2 mg calcium, and 3.40 mg vitamin C [27]. Each 100.0 g *H. megalanthus* fruit pulp contains 22.90 mg GAE total phenolic content, 13.07 g carbohydrates, 1.27 g fiber, 0.10 g of fat, 0.40 g protein, 17.99 mg phosphorus, 98.41 mg potassium, 11.73 mg calcium, 16.09 mg magnesium, 1.43 mg sodium, 4.35 mg zinc, 21.07 mg iron, 0.20 mg niacin, 5.93 g total sugar, and 11.34 mg of vitamin C [28], as well as volatile compounds such as alcohols, terpenes, paraffins, acids, esters, ketones, and odor-active compounds that influence flavor [29].

### 2.1. Betalains

Betalains are hydrophilic nitrogen-containing pigments abundantly found in red pitaya. They are classified as betaxanthins, which are yellow-orange in color, and betacyanins, which are red-violet in color, and these pigments are capable of absorbing 478 and 538 nm of light, respectively. Betalains have carboxylic functional groups rather than hydroxyl functional groups, in contrast to anthocyanins [34]. Betalains have multiple functions as antioxidants, anti-inflammatory, antiproliferative, hypoglycemic, radioprotective, cardioactive, diuretic hypolipidemic, neuroprotective, and osteoarthritis pain relievers [35,36,37].

### 2.2. Betalain Biosynthesis

In plants, arogenic acids originate from the _L_-tyrosine amino acid. Three key enzymes, i.e., tyrosinase, 4,5-DOPA-extradiol-dioxygenase, and betanidin-glucosyltransferase, are reported to be involved in betalain biosynthesis. Tyrosinase begins betalain biosynthesis by the hydroxylation reaction of _L_-tyrosine to _L_-DOPA, catalyzed by cytochrome P450 enzymes. Then, _L_-DOPA is cleaved by a ring-opening oxidation reaction by the enzyme 4,5-DOPA-dioxygenase to produce an intermediate, 4,5-seco-DOPA, which then produces betalamic acid by spontaneous intramolecular condensation [38]. As an alternative, a cytochrome P450 enzyme catalyzes the oxidation of _L_-DOPA to dopaquinone, followed by cyclization to form *cyclo*-DOPA [39,40]. The spontaneous conjugation of betalamic acid with the imino group of *cyclo*-DOPA results in the production of reddish-violet betacyanins [41]. Betalamic acid can also spontaneously condense with the amino group of amino acids to produce yellow betaxanthins. Betacyanins can have additional moieties, such as glucosyl or acyl groups, added to them enzymatically. Glycosylation takes place either before condensation of *cyclo*-DOPA with betalamic acid catalyzed by *cyclo*-DOPA 5-*O*-glucosyltransferase or after condensation of *cyclo*-DOPA with betalamic acid catalyzed by betanidin glucosyltransferase. Betacyanins can go through various enzymatically catalyzed changes in addition to glycosylation, adding a variety of moieties and enhancing the structural variety of betalains.

Recent studies in pitaya have made great progress in understanding betalain biosynthesis by exploring the key roles of transcription factors such as HmoWRKY42 [18], HuMYB132 [14], and HubHLH159 [13] that bind to the promoter regions of *HmocDOPA5GT1*, *HuADH1*, *HuCYP76AD1–1*, and *HuDODA1* and influence betalain biosynthesis.

## 3. Biological Activities

Pitaya consumption has multiple health benefits, and some of the biological activities of pitaya are summarized in Table 2.

### 3.1. Antioxidant Activities

Research shows that phenolic compounds provide protection against oxidative stresses [42,66] and act as antimutagenic, antitumor, and antioxidant agents [67]. Phenolics are prolific plant components that are predominantly derived from phenylalanine via the phenylpropanoid pathway [68]. Briefly, previous studies show higher ascorbic acid and total phenolic acid contents with free radical scavenging activity in the pulp of *H. undatus* and *H. polyrhizus* compared to the peel and pulp. These results were attributed to the polyphenol and ascorbic acid contents of the pulp [24]. Another study reported that the antioxidant capacity of different pitaya genotypes was interlinked with the level of betalain, its derivatives, and phenolic compounds, specifically acetyl coumarin and gallic acid [43]. The method of extraction also influenced phenolic compounds and antioxidant activity. In *H. undatus* pulp, phenolic compounds are higher and perform better in scavenging activity in ethanol extract (179.348 mg/L), followed by methanol (160.87 mg/L) and aqueous extraction (157.609 mg/L) [44]. Moreover, *H. undatus* fruit peel also has a strong antioxidant potential and can be used as a nutraceutical due to the high levels of polyphenolic compounds released with sonication for 45 min, an ultrasonic density of 32 W/L, a 6 M sodium hydroxide (NaOH) solution, and a liquid material ratio of 30 mL/g [45]. The methanolic extract of *H. undatus* shows 246 µg/mL phenolic contents and antioxidant activity with a half-minimal inhibitory concentration of 193 µg/mL [46]. Betacyanin fractions from *H. polyrhizus* fruit peel exhibit strong radical scavenging and reducing potentials. The results indicate that pulp is a good source of antioxidants, and peels are useful for nutraceuticals [47,48]. The difference in the observed results of antioxidant activity may be due to the analysis of different pitaya cultivars, seasonal variation, geographical locations, and experimental methods for detection [43,49].

Betanin, phyllocactin, and betanidin isolated from *H. polyrhizus* have peroxyl and nitrogen radical scavenging activity indicative of strong antioxidant capacity, provide defense against oxidative stress [50], and act as strong reducing agents [36]. Phyllocactin forbade H_2_O_2_ DNA damage in HT-29 cells at 15 µM, as detected by single-cell gel electrophoresis assay. Furthermore, phyllocactin treatment of Huh7 cells activated the transcription factor Nrf2, which increased PON1 transactivation, HO-1 protein levels, and cellular GSH. These findings indicate strong evidence for pitaya phyllocactin’s role in free radical scavenging and as a regulator of endogenous cellular enzymatic antioxidant defense [35].

### 3.2. Anticancer Activities

Oxidative stress induced by excess oxidants damages protein and DNA and increases cancer risk, while nutrients abundantly found in pitaya fruits, such as betalains, polyphenolics, minerals, vitamins, unsaturated fats, and tocopherols, provide chemoprotection and strong anticancer activities [48,51].

Luo et al. [51] extracted β-sitosterol, β-amyrin, and stigmast-4-en-3-one from *H. polyrhizus* and *H. undatus*, which possess cytotoxic effects on Bcap-37, PC3, and MGC-803 cancer cells. The three cancer cell lines displayed concentration-dependent antiproliferative effects with inhibitory concentration values of 0.64 and 0.61, 0.47 and 0.45, and 0.73 and 0.43 mg/mL, respectively, which indicate *H. polyrhizus* is a stronger inhibitor of MGC-803 cells than *H. undatus*. The results were attributed to the pentacyclic triterpenoids and steroids in pitaya peel extracts, which have anticancer properties [51]. *H. polyrhizus* peel extract had a higher IC_50_ (25.0 mg) than the flesh extract for cancer cell growth in B16F10 melanoma, which indicates that pulp is a good source of antioxidants and peels are useful for nutraceuticals [48].

### 3.3. Antimicrobial Activities

*H. polyrhizus* and *H. undatus* peel chloroform extracts exhibit strong antibacterial actions against *Bacillus cereus*, *Listeria monocytogenes*, *Staphylococcus aureus*, *Salmonella typhimurium*, *Enterococcus faecalis*, *Escherichia coli*, *Klebsiella pneumonia*, *Yersinia enterocolitica*, and *Campylobacter jejuni* in broth micro-dilution and disc diffusion methods. All extracts also prevented the development of all bacteria with minimal inhibitory concentrations between 1.25 mg/mL and 10.0 mg/mL [52]. Ismail et al. [53] identified oxygenated terpenes like 5-cedranone, eucalyptol, and α-terpineol from the methanolic extract of *H. polyrhizus* fruit that displays strong antimicrobial activity against bacterial and fungal strains of *Candida albicans*, *Pseudomonas aeruginosa*, *S. aureus*, *Aspergillus niger*, and *Fusarium oxysporum* [53]. Higher polyphenolic contents from the flesh and peels of *H. polyrhizus* fruit were extracted by implying an extract fractionation process and the contents inhibited the growth of all food-borne pathogens, yeast, and mold pathogens. The results indicate antimicrobial activity was widespread in the flesh and peel of pitaya [47].

### 3.4. Antihyperlipidemic and Antidiabetic Activities

Vegetable and fruit consumption (5–7 servings/day) reduces the occurrence of dyslipidemia, coronary heart disease, insulin resistance, and atherosclerosis, and this might be due to the antioxidants, vitamins, fiber, and other nutritional compounds in them [69]. A previous study on hypercholesterolemia-induced rats after a daily supplementation of 0.50%, 0.87%, and 1.17% of *H. polyrhizus* fruit for 5 weeks showed decreased total plasma cholesterol levels (49.14%, 56.72%, and 59.06%), as well as levels of triglyceride and low-density lipoprotein cholesterol while raising high-density lipoprotein cholesterol. These results indicate that *H. polyrhizus* fruit protects against dyslipidemia and cardiovascular disease [54] and improves cholesterol metabolism [70]. Another study on rats also found that *H. polyrhizus* fruit juice effectively decreased fructose-induced hypertriglyceridemia, atherosclerosis, and insulin resistance in rats, concluding that the anti-insulin resistance effect could be due to soluble dietary fiber, rich polyphenols, and antioxidant contents [55]. *H. undatus* fruit oligosaccharide intake may help overweight and diabetic people by reducing insulinemia and calorie intake [56]. *H. undatus* fruit juice had α-amylase and lipase inhibitory activity at 25–100 mL concentration in starch–agar gel diffusion and rhodamine agar plate assays, respectively [57].

### 3.5. Wound-Healing Activities

*H. undatus* stems and flower aqueous extracts applied to the surface of wounds in diabetic rats exhibit significant wound-healing properties. The healing effect is due to the extract’s increased DNA collagen content, hydroxyproline, tensile strength, total proteins, and better epithelization. That is why pitaya has been used in traditional medicine for the treatment of injuries [58]. Temak et al. [59] used burn-injured mice to assess the in vivo antibacterial activity of extracts from the peel of *H. polyrhizus*. In addition to its wound-healing properties, the extract exhibited a synergistic inhibitory effect with chloramphenicol on the growth of *P. aeruginosa* [59]. Tsai et al. [60] also reported the wound-healing properties of ethanolic extracts of *H. polyrhizus* stem, peel, and flower [60].

### 3.6. Anti-Anemia and Anti-Inflammatory Activities

Red pitaya *H. costaricensis* juice had a significant impact on pregnant women’s hemoglobin and erythrocyte levels on the seventh day of the intervention. The result was attributed to pitaya juice’s high iron contents, which protect against anemia [61].

### 3.7. Micro-Vascular Protective Activities

Two newly identified triterpenes Taraxast-20-ene-3α-ol (C_30_H_50_O) and taraxast-12,20(30)-dien-3α-ol (C_30_H_48_O) in the stem of *H. undatus* display a protective effect by increasing the vascular permeability of rabbits’ skin. They exhibit 53.5% and 70.1% reductions in Evans blue leakage, respectively, at 50 mg/kg, while troxerutin (64.5%) is indicative of the decreased permeability and raised capillary resistance of the two compounds [62]. These results suggest that pitaya stems can also be utilized in folk medicine due to the protective micro-vascular effect.

### 3.8. Hepato-Protective Activities

*H. polyrhizus* fruit consumption provides protection against liver injury. In a previous study, methanolic extracts of *H. polyrhizus* fruits significantly protected the liver against carbon tetrachloride-induced hepatotoxicity in rats in comparison to silymarin. The result was attributed to the phenolics and tocopherols in pitaya, which lower oxidative stress that causes liver damage [63]. Another study shows that *H. polyrhizus* juice supplementation for 8 weeks in high carbohydrate and fat diet rats resulted in increased aspartate transaminase, lower alanine transaminase, and alkaline phosphatase [64]. This suggests that *H. polyrhizus* juice may protect the liver due to its synergistic bioactive components, such as flavonoids, polyphenols, amino acids, alkaloids, vitamins, and steroids, which reduce paracetamol-induced hepatotoxicity in rats [64].

### 3.9. Prebiotic Effects

*H. undatus* and *H. polyrhizus* fruit flesh ethanol-extracted oligosaccharides (86.2 g/kg and 89.6 g/kg) trigger the growth of bifidobacteria and lactobacilli and show resistance to artificial human α-amylase and human gastric juice, giving maximum hydrolysis of 34.88% and 4.04%, respectively [56]. *H. polyrhizus* flesh has more prebiotic oligosaccharides compared to *H. undatus* [65].

## 4. Uses

Pitaya is an economically important cactus plant that has multiple uses for mankind. There is a lot of evidence available that reveals the multifaceted use of pitaya in different aspects of life, folklore, and mythology. Pitaya fruit can be eaten raw or processed into juice, wine, spreads, or desserts, as well as used in traditional herbal medicine [71]. Despite being valuable, pitaya was mostly an ignored and undervalued plant. However, there is currently a rapidly growing body of research regarding the benefits of pitaya for humans, including its significance in treating inflammation [72], cancer [73], diabetes [74], and natural colorants [75]. Additionally, the peels of pitaya also have a high capacity to absorb toxins [52,53].

### 4.1. Industrial Uses

Only pitaya is commonly grown for its high betalain content. Pitaya betalain is a natural edible, water-soluble pigment that is used in food products without affecting food flavor and has the additional benefits of rich protein, fat, fiber, and antioxidant activity [76]. As a result, the *H. polyrhizus* plant is now commonly used as a primary source for betalain extraction [75]. A total of 5% alcohol air residues from pitaya peels show similar viscosity potential to the 1% commercial thickener, indicating the potential of the use of pitaya peel as a thickener agent [77]. Additionally, skin products are formulated with the use of synthetic colors and chemicals that cause diverse skin allergic effects; recently, pitaya betalain has gained popularity as a natural colorant in cosmetic products with multiple skin benefits and no side effects [78,79].

### 4.2. Essential Oil

*H. undatus* and *H. polyrhizus* have high quantities of seed oil (18.33–28.37%) and total tocopherol contents (36.7 and 43.5 mg/100 g), respectively [49]. Previous studies show that pitaya seeds have a higher concentration of linoleic acid compared to canola, linseed, sesame, or grapevine. *H. undatus* and *H. polyrhizus* seed oil extracts contain 50% essential fatty acids with 48.5% linoleic acid and 1.5% linolenic acid [80]. Another study reported 660, 540- and 480 g/kg linoleic acid in *H. megalantus*, *H. undatus*, and *H. polyrhizus*, respectively [81]. Due to its high percentage of functional lipids, pitaya seed oil has the potential to be a new source of essential oils.

### 4.3. Other Uses

*H. polyrhizus* fruit wine contains a lot of aroma components, including 18 esters (66.17%), 12 alcohols (18.16%), 11 alkanes (4.32%), seven acids (5.94%), one aldehyde (0.09%), two olefins (0.09%), and three other volatile substances (0.23%) [82]. The addition of *H. undatus* or *H. polyrhizus* pulp to yogurt increased the amount of lactic acid, total phenols, antioxidant activity, and fermentation rate of milk [30]. Wheat flour cookies incorporated with 15% pitaya peel flour had higher ash, fiber, carbohydrate content, diameter, and spread ratio, indicating that pitaya peel flour can partially be substituted into wheat flour cookies to improve nutritional quality [83]. *H. polyrhizus* peel powder can be used as a fat substitute in ice cream for those on a calorie-reduced diet [84].

## 5. Taxonomy and Systematics of the Genus *Hylocereus*

Genus *Hylocereus* belongs to the Tracheophyte phylum, Magnoliopside class, Caryophyllales order, and Cactaceae family, with many species such as *H. calcaratus*, *H. costaricensis*, *H. escuintlensis*, *H. extensus*, *H. guatemalensis*, *H. megalanthus*, *H. minutiflorus*, *H. monacanthus*, *H. ocamponis*, *H. setaceus*, *H. stenopterus*, *H. triangularis*, *H. tricae*, and *H. undatus*. However, *Hylocereus* is a synonym, while *Selenicereus* is the accepted name after a phylogenetic study of Cactaceae that found no monophyletic relationship in genus or tribe between *Hylocereus* and *Selenicereus* in 2011 [85]. Similar results were found in another study in 2017, and the former genus *Hylocereus* nested species were moved to *Selenicereus* [86]. There are currently a total of 31 accepted *Selenicereus* species in the *Plants of the World Online* database [87].

**Figure 1:**
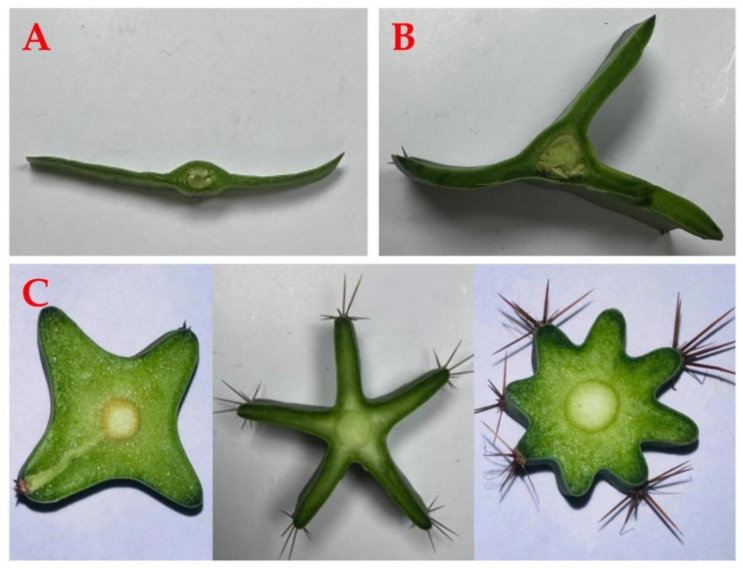
Number of ribs differentiated between the genera *Epiphyllum* (**A**), *Hylocereus* (**B**), and *Selenicereus* (**C**).

**Figure 2:**
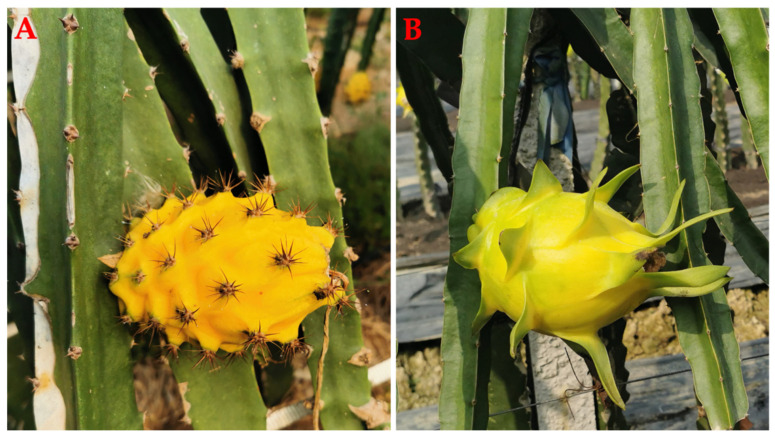
Two types of yellow peel pitayas. (**A**) *H. megalanthus*; (**B**) *H. undatus*.

The dicotyledonous family Cactaceae comprises between 120 and 200 genera, consisting of between 1500 and 2000 species. There are 16 species of *Hylocereus*, which have creamy white flowers, except *H. extensus* and *H. stenopterus*, whose petals are rose pink and red [88]. Three different genera comprise the cacti. *Hylocereus* produces shoots with three ribs, *Epiphyllum* produces shoots with two ribs, and the *Selenicereus* genus produces shoots with four ribs or more (Figure 1) [89,90]. *H. megalanthus* is tetraploid genotype, and all others are diploids [91,92]. In the scientific literature, *H. megalanthus* also appears under three names: *S. megalanthus*, *S. vagans*, and *Mediocactus coccineuss* [93,94]. Recent taxonomic and molecular data suggest that this species belongs to the *Hylocereus* genus; hence, the new name is *H. megalanthus*. Yellow peel pitaya has two types: the real *H. megalanthus* with spines on the peel and the yellow clones of *H. undatus* without spines on the peel, known as “golden” pitaya (Figure 2) [89,94,95].

## 6. Botany

### 6.1. Vegetative Growth

**Figure 3:**
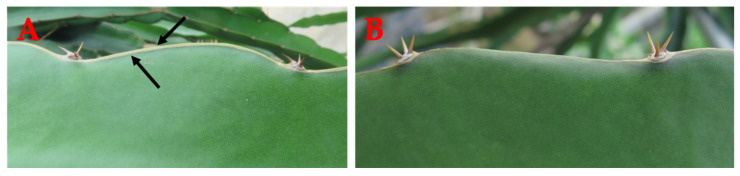
Brown strip at corner of ribs: stem difference between *H. undatus* and *H. polyrhizus* species. (**A**) *H. undatus*; (**B**) *H. polyrhizus*. Black arrows point to the brown strip.

Pitaya is a fast-growing, perennial, succulent, and climbing cactus plant. The cladode is green in color with some yellow, succulent, fleshy, pliable, and triangular in shape (3, 4, or 5 sides) with many areoles containing 2–5 short (1–3 cm) spines arranged on the scalloped edges [23]. Cladodes have stomata that are closed during the day and open at night [96]. These areoles act as buds and produce new stems and flowers. The stem is hairless, long, and narrow and can reach up to 20 feet. Pitaya stems produce adventitious roots that allow them to climb and creep. The stem under the soil produces roots that seek out nutrients [97]. *H. undatus* has a brown strip at the corner of the ribs, while *H. polyrhizus* has no brown strip (Figure 3).

### 6.2. Flowers and Phenology

**Figure 4:**
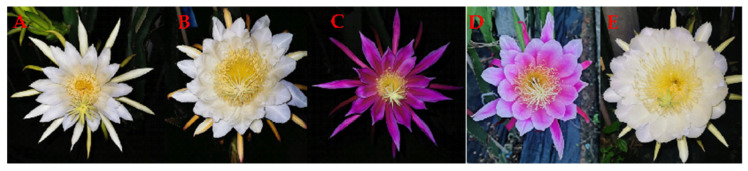
Flower phenotypes of different *Hylocereus* spp. at full-bloom stage at night. (**A**) *H. undatus*; (**B**) *H. polyrhizus*; (**C**) *H. stenopterus*; (**D**) hybridization offspring of *H. polyrhizus* and *H. stenopterus*; (**E**) *H. megalanthus*.

Pitaya flowers are nocturnal, bell-shaped, and very fragrant (musk aroma). They have yellow stamens and white, red, or pink petals with a prominent style (Figure 4). The length of a mature floral bud of a pitaya measures 20–36 cm, while its breadth measures 12–23 cm; its style length is 18–30 cm; the number of anthers is 1100–1195; the number of stigma lobes is 12–18; the length of stigma lobes is 2.0–3.5 cm; the length of the ovary is 4–8 cm; the availability of nectar is 4–9 mL; and each plant blooms one to seven flowers [98]. Pitaya flower is bisexual; however, some cultivars of both *H. undatus* and *H. polyrhizus* are self-compatible, while others are self-incompatible and need pollinators [99]. Buds at the distal end develop into light green, cylindrical floral buds in around 13 d and reach anthesis in 16–17 d. The flowers start opening at 8:00 p.m. in summer and 7:30 p.m. in autumn and reach a fully open stage at 12:00 p.m. in summer and 11:30 p.m. in autumn; pollination is completed around 2 a.m. and wilting lasts until the next morning [100].

### 6.3. The Fruit

**Figure 5:**
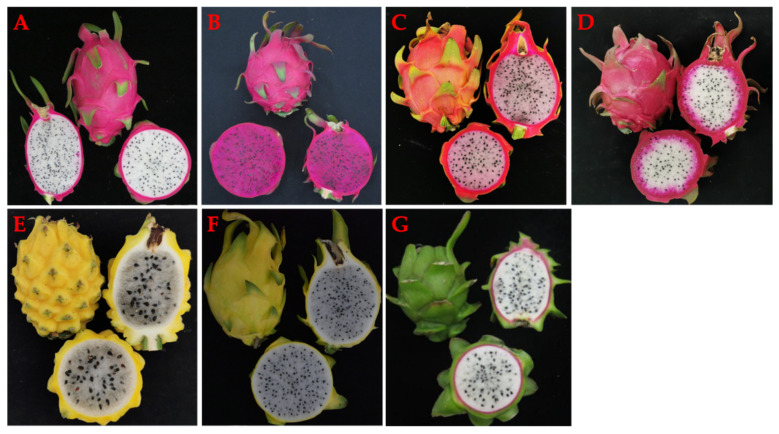
Fruit peel, pulp, and scale and areoles phenotype found in different *Hylocereus* species. (**A**) *H. undatus*; (**B**) *H. polyrhizus*; (**C**,**D**) hybridization offspring of *H. polyrhizus* and *H. undatus*; (**E**) *H. megalanthus*; (**F**) Golden pitaya (yellow clone of *H. undatus*), (**G**) *H. stenopterus*.

Pitaya fruit is round and oblong, with red (*H. polyrhizus*, *H. undatus*), green (*H. stenopterus*), yellow (*H. megalanthus*) peel, and the yellow clones of *H. undatus* without spines on peel having green scales (Figure 5) [101]. The length of pitaya fruit measures 10–20 cm, while its width measures 7–12 cm. Its girth is 10–18 cm, scale number is 10–35, scale length is 2.0–7.5 cm, peel thickness is 2–4 mm, weight of fruit is 220–840 g, pH value is 4.6–5.5, brix value is 12–18%, and time from pollination to fruit ripening is 30–50 d. In general, the fruiting season runs from May through January and occasionally into February. During fruit ripening, the green peel turns red/yellow except for *H. stenopterus*; after anthesis, harvesting begins 30–35 d for *H. polyrhizus*, *H. undatus*, and *H. stenopterus* in summer and 40 or more days in autumn, while 3–4 months for *H. megalanthus* in summer [98,102,103].

### 6.4. Post-Harvest

Pitaya fruit is non-climacteric and graded into 1st (500 g), 2nd (380 to 500 g), 3rd (300 to 380 g), 4th (260 to 300 g), and 5th less than 260 g [32] and stored in perforated bags for 14 d at a temperature of 14 °C, while 6 °C for 2 weeks can cause chilling injury when transferred to room temperature [103], except for yellow pitaya *H. megalanthus* [104]. Flesh translucency, wilting, softening, browning of outer flesh, darkening of scales, and poor flavor are symptoms of fruit chilling injury. Early harvested fruits 25 d from flowering are more sensitive to chilling injury compared to fruits harvested 30 to 35 d from flowering [103]. Ethanolic extract of propolis at 0.50% concentration maintains the storage life of pitaya fruit for 20 d at 20–22 °C and 80–85% relative humidity (RH) [105].

## 7. Pollination and Pollinators

Several pitaya cultivars are self-incompatible, so manual cross-pollination improves fruit set, size, and development [106,107,108]. *Hylocereus* flowers are large, making manual pollination easy. Manual pollination occurs from 11:00 p.m. to the next morning. Manual pollination yields high-quality fruit [108]. Pinching the bulging portion opens the flower for manual pollination. This exposes the stigma with pollens, which are brushed onto the stigma. Furthermore, anthers can be directly deposited on the stigma with slight finger pressure. Two flower pollens are enough for 100 flower brushes to pollinate. The pollen can be stored for 3–9 months at −18 °C to −196 °C. However, fruits produced after pollination with stored pollen at 4 °C are very small [109]. Some cultivars from *H. undatus* are self-incompatible, while some cultivars from *H. polyrhizus* and *H. monacanthus* are self-compatible. As a result, studies show that cross-pollination in and between species significantly increases fruit set and weight [98,110]. Bees, ants, wasps, and moths are biological pollinators of pitaya in the evening [98].

## 8. Cytology

Cytological observations show that *H. undatus*, *H. triangularis*, *H. guatemalensis*, *H. monacanthus*, *H. polyrhizus*, *H. ocamponis*, *H. triangularis*, and *H. trigonus* are diploid (2n = 22), whereas *H. megalanthus* is tetraploid (2n = 44) (Table 3) [90,92]. Fruit size, number of seeds, and pollen viability are negatively impacted by abnormal chromosomal disjunction at anaphase I in *H. megalanthus* pollen mother cells [92]. Natural hybridization between two diploid taxa that are closely related to one another produced *H. megalanthus* (2n = 44). Hybrids of all possible ploidy levels are produced by crossing tetraploid *H. megalanthus* with different *Hylocereus* genotypes [17,94].

## 9. Agronomy, Cultivation, Pests, and Diseases

### 9.1. Agronomy and Cultivation

Pitaya grows in regions with tropical and sub-tropical climatic conditions with warm, moist, sandy, rich organic matter, well-draining, and pH level neutral to acidic soil (7.0–5.8) [111]. In tropical and sub-tropical regions, salinity is a major problem, resulting in delayed growth and development of pitaya plants [112,113]. Therefore, acidic soil can be treated with ferrous sulfate, and rearing basic soil with chelated iron maintains healthy and strong pitaya plants [114]. To control soil acidity and improve pH, the addition of 1.8 t/ha of limestone improved soil base saturation, pH, calcium, and magnesium while decreasing potential acidity, facilitating growth, nutrition, and production of *H. monacanthus* [115]. Pitaya plants in dry regions need mulching to maintain soil moisture. A study shows pitaya performs better in natural grass mulch compared to black fabric, coconut chaff, and purslane mulch [116]. Natural grass mulch has a higher bacterial and lower fungal abundance than black fabric mulch. However, both natural grass and black fabric mulch have pros and cons with regard to the cultivation management of pitaya orchards [117].

#### 9.1.1. Irrigation

Pitaya is an attractive crop for farmers in semi-arid and arid regions owing to its drought resistance. However, it needs 25–50 inches of annual rainfall or careful water management through twice-weekly irrigation because, during fruit development, uneven soil moisture may lead to fruit splitting [111,118]. However, excessive watering can cause root rot, fungal disease, and fruit drop. Precocious flowering in pitaya is induced by refraining from watering in the early spring [118].

#### 9.1.2. Fertilization

Pitaya plants at 2–3 months need no fertilizer; at 1 year, they need 4 ounces of inorganic fertilizers at a 6-6-6 or 8-3-9 ratio from early spring to winter, along with 4 pounds of manure or compost per plant in summer; at 2–3 years, need 5 to 6 ounces of inorganic fertilizers at the same ratio, along with 6 pounds of manure or compost per plant in summer; and at 4 or move years, need 8 to 12 ounces of inorganic fertilizers, along with 5 pounds of manure or compost per plant in both spring and mid-summer [111,119,120]. Some studies suggest 1.2 kg fertilizer ratio of N:P:K:Mg (9.6:4.8:17.6:2.4) and 12 kg compost per plant per year [121], 45 t/ha organic fertilizer +3% biochar [122], 44–67 mg/dm^3^ of phosphorus in the soil [123], 170–300 g of nitrogen per plant [124], 9 kg of steer manure with 3.5 ounces of 13-13-13 fertilizer/plant [111], and N-P-K at 46-0-0, 24-24-0 and 16-16-16 [125] to pitaya plants.

#### 9.1.3. Light

*H. polyrhizus* is susceptible to the harsh environment of sunburn; in such a case, it is grown under black shade. In dry or hot 37 °C conditions, pitaya needs full sun to mild shade or 50–75% black shade cloth to avoid sunburn [126], but too much shadow may impair fruit output and quality. A lighting supplementation of 15–25 days in Vietnam regulates flowering in pitaya, while in Taiwan, it takes four weeks in late fall (mid-October to mid-November) and three months in winter and early spring (January to March) [127].

#### 9.1.4. Temperature

Pitaya thrives at 18–27 °C, but an extreme temperature of 39 °C is not suitable [128]. Cold damage is the biggest issue in northern China [129,130], while a humid climate causes pests and diseases in pitaya produced in Malaysia [131,132]. A study reported that fresh-cut pitaya fruit storage at 5–10 °C increased the accumulation of total soluble phenolics reactive oxygen species and improved antioxidant activity [133,134]. For the purpose of floral buds induction in pitaya, a 29–32 °C day and 19–22 °C night temperature is required during long days [128]. 

#### 9.1.5. Pruning

Pitaya is a climbing cactus that requires a trellis or other means of support. The first stage of pruning includes training the growing plants, eliminating any lateral stems along the main stem until they reach the trellis, and finally tying the main stem to a trellis. Tips should be cut to induce branching. Pitaya plants need regular pruning with a clean tool to avoid fungus and insect infestation, balance plant weight on the trellis, increase light penetration into tangled middle stems, and induce flowering. Pitaya pruning involves trimming back long, broken, tangled, or dead stems once annually in young plants and three times in mature plants [23,135]. A study on *H. undatus* pruning shows that cane pruning induces maximum flowering and produces new shoots as compared to spur, spur–cane combined, and sanitary pruning [136]. Another study further reported improved yield characteristics of pitaya with cane pruning, leaving 12 and 15 cladodes per meter on a trellis [137].

#### 9.1.6. Flowering

Pitaya plants developed from seeds require 2–3 years to first flower, while cuttings take 1–2 years. In southern China, pitaya flowers in 10–15 cycles from late April to mid-October, depending on species, climatic conditions, and management. The normal flowering season of pitaya in Taiwan is from June to October. Additionally, supplementation with 4–6 h of light using a 15-watt LED at a 4–5 ft distance hanging 1–2 ft above plants is used to induce off-season flowering [16,98,111].

Pitaya flowering involves multiple regulatory genes, such as the Krebs cycle of glucose metabolism; sucrose-, indole acetic acid (IAA)-, ethylene (Eth)-, abscisic acid (ABA)-, jasmonic acid (JA)-, brassinosteroid (BR)-, gibberellin (GA)-synthesis- and response-related genes; sepallata; constans-like, *FLOWERING LOCUS T*, *LEAFY*, circadian rhythm-associated, and *HD3a*-like transcription factors; *CYCLING DOF FACTOR*, *AGAMOUS*, and *TCP* genes. These results show that pitaya flowering is modulated by a complex regulatory network, including hormone biosynthesis and signaling [16,138]. Khaimov and Mizrahi [139] found that *H. undatus* and *H. megalanthus* exposed to N-(2-Chloro-4-pyridyl)-N’-phenylurea, a synthetic cytokinin, which promoted precocious flowering, whereas GA_3_ delayed flowering and decreased total flower yield. Some studies also indicate that the flowering of *H. undatus* and *H. polyrhizus* is inhibited by high temperatures (38 °C), while moderate temperatures (32–34 °C) encourage flowering [140]. It has been reported that foliar application of mono-potassium phosphate (1% *w*/*w*), Folar-K^®^ (0.1% *v*/*v*), potassium nitrate (1% *w*/*w*), and Box-Flower^®^ (1% *v*/*v*) on a weekly basis for three months induces pitaya flowering by 18.7, 27.7, 33.2, and 38.0%, respectively [141,142]. Ye et al. [143] found *HuNIP6*;*1* may be involved in pitaya floral opening.

### 9.2. Pest and Diseases

Pitaya stems are succulent and susceptible to various fungi such as *Alternaria alternata* [144], *Aureobasidium pullulans* [145], *Neoscytalidium dimidiatum* [146,147], *Colletotrichum gloeosporioides* [148,149,150], *Bipolaris cactivora* [151,152,153], *Nigrospora sphaerica* [154], *Gilbertella persicaria* [155,156], *Botryosphaeria dothidea* [157,158], *Curvularia lunata* [159], *Fusarium solani* [160], *Fusarium proliferatum* [161], *Aspergillus flavus*, *Fusarium lateritium*, and *Aspergillus niger* [32,162].

Different fungicides and techniques have been reported to disinfect pitaya fruits, such as emulsions of *Cinnamomum zeylanicum* and *Eugenia caryophyllus* at concentrations of 500 and 1000 µg/mL, which reduced fungal growth up to 30–31% [144], submicron chitosan dispersions at 1.0% with 600 nm droplets [163,164], as well as the application of 3 mM ferrous ion (Fe^2+^) and benomyl and copper oxide chloride [32,162]. *Cactus virus X* [165,166,167,168], *Schlumbergera virus X* [169,170], *Zygocactus virus X,* and *Pitaya virus X* [170,171,172] have been reported in pitaya stems. Pitaya bacterial pathogens include *Enterobacter cloacae* and *Erwinia chrysanthemi*, which cause bacterial soft rot [173,174,175]. Pitaya plants are severely damaged by the *Aphis gossypii*, *Spodoptera litura*, and *Cactophagus spinolae* pests [176,177,178]. Pitaya treated with hot air at 46.5 °C for 20 min is used to disinfest fruit from insects [179].

## 10. Propagation, Micropropagation, Cell, and Tissue Culture

### 10.1. Conventional Propagation

Conventional propagation refers to sexual or asexual approaches to plant propagation. The sexual method includes growing plants using seeds, while the asexual approach includes cutting and grafting. ElObeidy [180] suggested pitaya from seed propagation followed on wet filter paper in a Petri dish or (1:1) peat moss and sand mix at 20–24 °C with 12 h light intensity of 0–500 lx. Andrade et al. [181] reported the use of 65 mL substrate for pitaya propagation from seeds. Kari et al. [182] showed *H. polyrhizus* seed propagation in modified MS Chinese A basal media with 0.5 ppm indole-3-butyric acid (IBA) and 1 ppm kinetin (KT) gives good results.

Propagation of pitaya through cuttings includes the selection of healthy, disease-free, and young juvenile cuttings from mother plants [183]. Cuttings of about 20–70 cm are prepared by removing two-thirds of the apical buds at the base; one-third of the apical buds are left intact at the top. A slanted cut is made at the stem base, and about 1–2 cm of the green tissues are removed to expose the stem base. The cuttings are brushed with calcium carbonate to sterilize them from fungus and bacterial infections. Pitaya stems are susceptible to telluric pathogens, so cuttings are kept in a clean, dry, and shady place for 3–7 d [97]. Well-drain substrate and compost at a ratio of 9:1 are mixed and irrigated, and excess water is left to drain. ElObeidy [180] suggests dipping the stem base in 10 mM IBA solution for better rooting. Next, the cuttings are inserted into the medium, about 2–4 cm deep. Pitaya needs support from a trellis system or frame in pots. The cuttings are attached with support, and weekly irrigation follows [97].

### 10.2. Tissue Culture

The micropropagation process of pitaya includes the establishment of an aseptic culture from shoot tips and lateral nodes, shot elongation, mass multiplication, and rooting. Micropropagation of pitaya is moderately difficult due to the release of polysaccharides and their succulent nature, resulting in fungus and bacterial contamination, and necrosis of explants [93,97,184]. Pitaya can be propagated in vitro with both direct and indirect regeneration methods, which makes it easy to move the germplasm to other laboratories without quarantine and phytosanitary issues.

#### 10.2.1. Selection and Preparation of Disinfectant Explants

Pitaya in vitro propagation needs strict consideration of pretreatment and dual disinfection treatments. Before selection of explant cuttings, pitaya plants are treated with 1.0 g/L fungus disinfectants benomyl three times weekly, followed by cutting 4–5 cm sections of young shoots, 30 min rinsing in tap water with one drop of nonionic surfactant Polysorbate 20 or Tween 20, and treated with 1.0 g/L fungicide benomyl solution for 5 min, then air dried for 20 min, surface sterilized by 70% *v*/*v* ethanol for 30 s, followed by washing one time in sodium hypochlorite 3% *v*/*v* solution and three times in distilled water. Then, they are sterilized under vacuum for 5 min with a biocide or antimicrobial plant preservative mixture 4% *v*/*v*, followed by 0.2% *v*/*v* sodium hypochlorite sterilization for 1 min and rinsing in distilled water three times. Finally, explants are trimmed to areoles within 1 cm of surrounding tissues [97,185].

#### 10.2.2. Basal Media In Vitro

Pitaya culturing needs 25 mL of Murashige and Skoog (MS) medium in 200 mL glass jars. Mohamed-Yasseen [185] reported basal media for *H. undatus* as 30 g/L sucrose, 8 g/L agar, 0.5 µM naphthaleneacetic acid (NAA), and 0.5 µM thidiazuron (TDZ), while recent Trivellini et al. [97] studies suggested 500 mg/L 2-(N-morpholino) ethanesulfonic acid, 30 g/L sucrose, 2.5 g/L gelrite, 300 mg/L glutathione, and 1 mL of 200 mg/L cefotaxime. Basal media for yellow pitaya *H. megalanthus* seeds in vitro propagation include 1.2 µM thiamine-HC1, 116.6 µM myo-inositol, 0.7% agar, and 3% sucrose [93]. The pH is adjusted to 5.8 using 1 M KOH, 0.1 M NaOH, or 0.1 M HCl and subjected to autoclaving for 15–20 min at 121 °C with a pressure of 98–120 kilopascals. The explant cultures are kept in 8 h dark and 16 h light conditions (40 µmol m^−2^s^−1^) at 22–24 °C [97,185].

#### 10.2.3. Shoot Proliferation

Intact apical meristems exhibit strong apical dominance, thereby excising 1–3 mm of apical meristem [93,185] or adding 5 µM 6-(γ,γ-Dimethylallylamino) or (2ip) in medium to remove the effect [186]. Shoot subculturing requires plant growth regulators such as auxin and cytokinin in fresh medium. Infante [93] reported maximum shoot thickness and proliferation rate with a combination of 0.54 µM NAA and 2.2 µM benzyladenine (BA) and 0.27 µM NAA and 4.4 µM BA, respectively, while higher shoot length was achieved with 0.05 µM NAA with 2.2 µM BA for decapitated and 4.4 µM BA for intact meristems [93]. Mohamed-Yasseen [185] observed the maximum number of shoots using 0.5 µM NAA with 0.05 µM TDZ and shoot length with 0.5 µM TDZ. Trivellini et al. [97] observed maximum shoot length with IBA at 0.25 mg/L and zeatin (ZT) at 3 mg/L. Bozkurt et al. [187] reported maximum shoot proliferation with 4.0 mg/L 6-BA and rooting with 1 mg/L IBA. Qin et al. [188] showed 5.5 mg/L 6-BA and 0.1 mg/L NAA, while in a recent study by Lee and Chang [189], 0.20 mg/L NAA, 200 mg/L activated charcoal and 1.0 mg/L 6-BA were the best media for shooting *H. polyrhizus*. Hua et al. [190] achieved a maximum number of shoots per explant with 3.0 µM ZT and 0.5 µM IBA, while the best shoot propagation media were 13.68 µM ZT and 2.46 µM IBA. Fan et al. [191] used *H. undatus* areoles in solid MS with 2.0 μM 6-BA and 0.5 μM NAA for shooting.

#### 10.2.4. Rooting and Acclimatization of Plantlets

Half-strength MS media with 0.5 mg/L NAA and 0.3 mg/L IBA [188] induce precocious rooting; however, if IBA is used in the shoot proliferation media, then maintaining shoots for 6–7 weeks on the media also allows rooting [97]. Plants that reach 2.5 cm in height are hardened in bottles for 4–5 d; roots are cleaned from agar using tap water, rinsed for 10 min in 10% (*v*/*v*) fungicide [188], and transplanted in plastic pots with autoclaved 1:1 perlite:soil. Orea and Medrano [192] suggested the use of tezontle and perlite mediums for good acclimatization and rooting. Pots are covered with plastic lids, maintained under the growth chamber 7–10 d, then transferred to the greenhouse and kept under a mist irrigation system for 4 s every 15 min [97,185].

## 11. Somatic Embryogenesis and Shoot Bud Organogenesis

Somatic embryogenesis has been reported by Infante [93], who observed that yellow pitaya cotyledon and roots supplemented with 2.7 µM or 5.4 µM NAA were effective for embryogenic callus induction. Karimi et al. [193] observed maximum callus frequency with 4 mg/L of NAA or 2, 4-dichlorophenoxyacetic acid (2,4-D) in the pitaya of the Peruvian apple cactus. In *H. undatus*, 4 mg/L 2,4-D induces maximum callus with embryogenic potential at 49 d [194].

Direct somatic embryogenesis from the cotyledon and cladodes of *H. megalanthus* and *H. polyrhizus* was achieved in 30 d with MS containing 2 mg/L of each KT and BAP [195]. Direct shoot organogenesis has been reported by Dahanayake and Ranawake [196] in stem and stem cuttings of *H. undatus* with a combination of 0.01 mg/L NAA and 2.5 mg/L BA, as well as subculturing in 0.01 mg/L NAA containing MS medium for rooting. *H. costaricensis* areoles with spines culturing on 30 µM BAP-induced callus formation. Pelah et al. [197] show indirect organogenesis from proximal parts of cotyledon by 200 µM TDZ and rooting 5.3 µM NAA.

## 12. Breeding through Biotechnology

### 12.1. Somaclonal Variation and In Vitro Selection

Tissue culture can be employed on long-term tissue to improve important genetic varieties and maintain the genetic fidelity of stocks. Somaclonal variation is the genetic variation found in tissue-culture-derived material. Tissues kept in non-differentiated form for a long time may increase the somaclonal variation compared to the natural mutation rate in plants [198]. The genetic variation in regrown plants from gametic or somatic cell culturing is an important source for breeding germplasm. Despite several somaclonal variation’s potential benefits, no major crop species have been commercially cultivated, considerably improved new varieties as a result of somaclonal variation. Stress-resistant tissue culture cell lines can be selected in vitro because tissue culturing is an extensively used technique for breeding, specifically in the selection of lines with stress tolerance. A stress-causing agent is subjected to tissue cultures containing dividing cells for selection. Adding selecting agents that will change other features of the phenotype is an effective way to acquire plants with desired qualities [199]. Fan et al. [191] used 442 inter-simple sequence repeat (ISSR) markers, producing 55 primers exhibiting no polymorphism and somaclonal variation after 15 multiplication cycles of shoots; the results were attributed to the existence of high genetic fidelity in *H. undatus*. Hua et al. [190] and Rodrigues et al. [200] also reported no somaclonal variation after multiple in vitro cycles.

### 12.2. In Vitro Mutagenesis

Deng et al. [201] reported mutagenesis by using 3.7–3.9% ethyl methanesulfonate for 8–9 h and an irradiation dose of 38–42 gamma rays with cobalt-60. Fourteen morphological mutagens of *H. polyrhizus* seedlings were confirmed by ISSR analysis, which displayed 67 amplified bands with 71% polymorphism and 0.01–0.41 genetic diversity; in comparison with the control, five mutants were noted as highly diverse.

### 12.3. Androgenesis, Gynogenesis and Altered Ploidy

The breeding period can be shortened by using haploids, which can be produced using in vitro androgenesis (anther culture or microspore culture). Additionally, chromosomal doubling can create homozygous diploid lines.

Benega Garcia et al. [202] cultured uninucleate stage anthers from *H. undatus*, *H. polyrhizus*, and *H. megalanthus* and, after 3 d of culturing, achieved a direct androgenesis response with and without BA/picloram in *H. megalanthus*, while 0.1 mg/L TDZ resulted in single direct androgenesis response. These androgenic embryos were subcultured, and haploid, monoploid, mixoploid, and dihaploid plantlets were achieved. These results were attributed to culture-, medium-, and species-dependent androgenesis in pitaya. Benega Garcia et al. [203] reported dihaploid and higher ploidy levels in *H. megalanthus* gynogenic plantlets by culturing unpollinated ovules at the uninucleate stage on TDZ/2,4-D and 0.18–0.26 M sucrose. These results were attributed to the sucrose- and species-dependent gynogenic responses in pitaya. Further, the origin of these gamete-derived lines was confirmed by SSR markers and flow cytometry [204]. Surprisingly, the haploid lines of *H. monacanthus* derived from androgenesis [202] passed through spontaneous genome doubling, ending in the dihaploid (2×) lines [204]. However, these dihaploid lines have poor vigor, abnormal flower, and aborted anthesis except single fruit set containing few viable seeds, which upon germination, produced normal plants similar to the donor. Fagundes et al. [205] adjusted the anther culture medium for *H. undatus* and *H. polyrhizus* without achieving haploid plants by 20 mL of a culture medium with sucrose 100 g/L, calcium 518–616 mg/L, boric acid 619–636 mg/L, agar 6 g/L, and pH 5–6.

Autotatraploid, autohexaploid, and autooctaploid lines were reported from *H. monacanthus*, S-75 hybrid, and *H. megalanthus*, respectively, by application of oryzalin and colchicine to axillary buds and seeds [17]. These lines show morphological differences with reduced fruit size, number of stomata, viable seeds, and viable pollens [206]. Autotatraploid and autohexaploid lines further show metabolomic differences in increased intermediates of TCA cycle, amino acids, flavonoids, organic acids, reduced sugars, and betacyanin contents compared to doner plants [207]. These results were attributed to the reduced fruit quality and weight of autopolyploidization, which is not a suitable method of breeding to obtain large fruits in pitaya.

### 12.4. Virus-Induced Gene Silencing in Pitaya

Virus-induced gene silencing (VIGS) is a powerful tool in plant molecular biology that can be used to study gene functions and analyze gene regulation. VIGS involves introducing a modified virus into a plant, which then triggers the plant’s own defense mechanism to silence specific genes. By targeting specific genes of interest, researchers can observe the effects of their silencing on the plant’s phenotype and better understand the gene’s function. In the case of pitaya, VIGS could be used to investigate the function of specific genes involved in traits such as fruit development, disease resistance, or nutrient metabolism. Chen et al. [13] attempted VIGS in a ‘Hongguan No. 1′ pitaya. *A. tumefaciens* strain GV3101 (pSoup-p19) containing recombinant vector pTRV2-HubHLH159 was injected into the scales of pitaya pericarp. Two weeks later, it was observed that the scales of ‘Hongguan No. 1’ did not turn red when *HubHLH159* was silenced, and betalain content at the injection site was significantly lower than that of the control, indicating that HubHLH159 could promote pitaya betalain biosynthesis. Through VIGS assays, HuMYB132 [14] and HuWRKY42 [18] were also found to be involved in the regulation of betalain biosynthesis in pitaya. As scientists continue to advance the understanding of pitaya genetics, VIGS could become a valuable tool in unraveling the complexities of this fascinating fruit crop.

## 13. Marker Technology

A variety of genotypes exist in pitaya with different production potential, fruit characteristics, ribs, and flowering. However, due to the recent domestication of pitaya, very little is known about the difference in genetic diversity, specifically the comparative analysis of pitaya accessions by a combination of DNA markers.

### 13.1. Morphological and Biochemical Markers

Abirami et al. [208] differentiated pitaya genotypes (*H. undatus*, *H. costariscensis*, and *H. megalanthus*) selected from the Nicobar and Andaman Islands based on morphological characteristics of fruit peel and pulp color, spine numbers at cladodes, areole length, rib margin, and stem wax. The highest variation among the observed species was observed in pulp weight, while the lowest variation was in the anthers distance from the stigma. Moreover, variations in biochemical markers, including phenol, flavonoid, carotenoid, β-carotene, xanthophyll, and scavenging activities distinguished tested pitaya genotypes. These results suggest the potential of *H. costariscensis* responsible for higher nutraceutical pigment production and potential use in future breeding.

Silva et al. [209] manually fertilized *H. undatus* with *H. polyrhizus* and *H. setaceus*. Morphological approaches determined genetic diversity in interspecific pitaya hybrids. Based on cladode characteristics (for 51 individuals of 45 progenies and six parents), clustering analysis by the UPGMA method assessed six characters such as stem length and diameter, areole distance, arch height, and spine number and size. Due to the significant variation in pitaya hybrids, eight hybrids showed improved traits that could be exploited in breeding programs. Based on morphological traits, Tao et al. [210] classified 50 pitaya accessions selected in China into three classes, separating wild, red pulp, and white pulp genotypes.

### 13.2. DNA-Based Molecular Markers

DNA-based molecular markers used in pitaya are summarized in Table 4.

#### 13.2.1. RAPD

Since a universal set of primers, no probe isolation, and no nucleotide sequencing are required for random amplification of polymorphic DNA (RAPD) analysis, it is commonly employed for studying genetic variation.

Tel-Zur et al. [211] reported the genetic variability between nine *Selenicereus* (*S. megalanthus*, *S. grandifloras*, *S. coniflorus*, *S. atropilosus*, *S. rubineus*, *S. macdonaldiae*, *S. wercklei*, *S. innesii Kimnach*, *S. murrillii*) and five *Hylocereus* (*H. undatus*, *H. ocamponis*, *H. costaricensis*, *H. purpusii*, *H. polyrhizus*) species in 34 total taxa using 173 RAPD markers. They identified two distinct groups, *Selenicereus* and *Hylocereus*, by dendrogram. Further, principal coordinate analysis (PCoA) revealed the separation of *H. megalanthus* from *Selenicereus* species, indicating that *H. megalanthus* is tetraploid while specific *Hylocereus* species are diploid. Thus, the species is regarded as a natural cross between *Hylocereus* and *Selenicereus*, and it may even belong to a different genus.

Junqueira et al. [212] reported genetic variability among 16 accessions of *H. undatus* in Brazil selected at Embrapa Cerrados germplasm resource that had different production capacities with 111 RAPD markers and found a genetic distance between 0.006 and 0.148 (45% polymorphism). The genetic distance of accesses 52 and 61, with 25 fruits per plant and none, respectively, show high variability. Junqueira et al. [213] further used 162 RAPD markers to test genetic diversity between 13 pitaya genotypes selected in Brazil and amplified the average of 11 bands per primer, showing 95% polymorphism and a genetic distance between 0.08 and 0.84. Additionally, Unai MG (*H. setaceus*) showed a high genetic distance when compared with other genotypes. The results were attributed to the presence of genetic variability in the same species.

Similarly, Rifat et al. [214] further explored genetic diversity by 43 RAPD markers in 15 *Hylocereus* genotypes selected from Bangladesh and observed 86.05% polymorphism and a genetic diversity of 0.327. Further, UPGMA analysis classified the 15 genotypes into three groups. Legaria Solano et al. [215] also used RAPD markers to test 50 pitaya germplasm collected from nine states in Mexico and one from Columbia. Polymorphism at 92% is attributed to the high genetic variability among different regions. The germplasm of Columbia was genetically identical to some of the Mexican germplasm, suggesting they have the same origin, while the germplasm of San Luis Patigo, Mexico, and Hidalgo region were genetically different from others, suggesting Mexico possesses diverse pitaya genotypes.

#### 13.2.2. ISSR

Abirami et al. [208] also used 16 ISSR markers to differentiate three *Hylocereus* genotypes (*H. undatus*, *H. costariscensis*, *H. megalanthus*) selected from the Nicobar and Andaman Islands. They observed a total of 178 bands, with 19 in UBC811 and 5 in UBC887, polymorphism between 20 and 92%, polymorphic bands between 1 and 13, and polymorphic information content (PIC) varying from 0.4 for UBC895 to 0.9 for UBC856. These ISSR markers divided the tested three genotypes into two groups based on geographic locations. Group 1 contains *H. megalanthus* and *H. undatus* with a genetic similarity of 52%, and group 2 contains only *H. costariscensis* with a genetic similarity of 76%.

Tao et al. [210] used 111 ISSR markers among 50 accessions of *H. undatus* and *H. polyrhizus* in China. Each primer of ISSR produced 4–10 with an average of 6.9 bands per primer and 66.1% polymorphism with UBC824, UBC891, and UBC900 primers effectively fingerprinting 50 selected genotypes. Polymorphism information content was between 0.4 and 0.9, which suggests a higher level of genetic diversity, and UPGMA analysis further divided the 50 accessions into two major clusters with a genetic distance of 0.23.

Morillo et al. [216] recently tested 76 *H. megalanthus* Columbian genotypes with eight ISSR markers and observed 225 alleles with 85–90 polymorphic loci. A heterozygosity rate of 0.34 and a genetic differentiation coefficient of 0.26 are attributed to high genetic diversity. These ISSR markers divided the 76 genotypes into three groups with 25% similarity based on geographic location, while some groups contain a mixture of each individual genotype. Hernández-Andrade et al. [217] characterized the genetic diversity of nine pitaya genotypes collected from central, west, and east parts of Mexico, exhibiting moderate genetic diversity, 25–53% polymorphic loci, and expected heterozygosity 0.07–0.13. Further, two genetic lineages were found, one in the central and west regions and another in the east and west regions, while the genetic differentiation of nine selected pitaya genotypes was similar to wild pitaya species.

#### 13.2.3. SSR

Among RAPD, ISSR, and amplified fragment length polymorphism (AFLP) markers, simple sequence repeat (SSR) leads by providing high polymorphism levels, multiple alleles, and co-dominance. Nashima et al. [218] used 16 SSR markers among the 32 accessions of *H. undatus* and *H. megalanthus* selected in Japan and found heterozygosity between 0.2 and 0.9%, polymorphism between 0.4 and 0.8, fixation index of 0.01, and outcrossing rate of 0.9. As a result of UPGMA analysis, five groups were produced according to their genetic diversities in fruit characteristics. Pan et al. [219] reported the use of SSR markers on 46 *Hylocereus* genotypes collected from China and abroad, and 52 effective alleles were amplified between 1.1 and 4.0 by 18 SSR markers that show a genetic similarity coefficient between 0.6 and 0.9. These accessions were divided into four groups based on pulp color and similar identities, with an 80% similarity index between groups. Li et al. [204] used 23 SSR markers to distinguish the *H. monacanthus* and *H. megalanthus* potential gamete-derived lines and donor species. It was observed that both the two donors and tetraploid regenerants from *H. megalanthus* exhibit five bands (1–5) at the SSR locus *pchi44*, whereas the di-haploid and double di-haploid lines exhibited two types of four-band patterns, namely, type 1 (1, 2, 3, and 5) or type 2 (2, 3, 4, and 5).

#### 13.2.4. AFLP

Pagliaccia et al. [220] selected 230 *Hylocereus* accessions and subjected them to 51 AFLP, resulting in the detection of seven main clades and 126 putative clones, with high bootstrap support of 96% in the wildtype accession, 94% in *H. megalanthus*, 83% in *H. ocamponis*, and 60% in *H. guatemalensis*. American Beauty and Bien Hoa Red were found in one clonal cluster of the *H. guatemalensis* clade. One accession of Halley’s comet was clustered in the *H. undatus* clade and one in *H. guatemalensis*, while the rest were in the hybrid clade. Vietnamese Giant and Mexicana were clustered in *H. undatus*. Lisa, Oregona, Cebra, and Rosa were found in the *H. polyrhizus* clade. Genotypic diversity within putatively named varieties was observed, although some of the differentially named varieties were identical. These results were attributed to the renaming, which happened due to the easy distribution of germplasm and propagation of cuttings.

Cisneros and Tel-Zur [91] investigated the genetic characteristics of 59 diploid, triploid, tetraploid, pentaploid, or hexaploid progenies of *H. monacanthus*, *H. undatus*, and *H. megalanthus* obtained by self-pollination interspecific homoploidy and interploidy using 192 AFLP markers. *Hylocereus* accessions and their progenies show 97.5% and 98.1% polymorphism, respectively, indicating high heterozygosity between species and hybrids. Further, *H. megalanthus* was identified as an unknown male progenitor of the allotriploid S-75 (*H. monacanthus* × *H. megalanthus*).

#### 13.2.5. SNP

Only two pitaya genomic maps have been created so far. Using whole genome re-sequencing, Wu et al. [221] reported the highest-density genetic map by 6434 polymorphic single polymorphism nucleotide (SNP) markers from 198 accessions of the F1 hybrids (*H. undatus* and *H. monacanthus*), and according to the chromosomal count, 11 linkage groups were successfully identified with length cover between 255.1 and 2070.07 cM. The entire length of the map was 14128.7 cM, with an average gap of 2.2 cM between each pair of 6434 markers. Genetic distances of 2070.07 cM (708 markers), 1986.08 cM (1340 markers), and 1954.33 cM (836 markers) separated the three largest linkage groups, LG01, LG04, and LG07.

Chen et al. [222] used a digestion-based genotyping-by-sequencing (GBS) approach in the genetic mapping of the F1 hybrids from *H. undatus* and *H. polyrhizus*, which comprised 203 accessions. *H. undatus* and *H. polyrhizus* are equally heterozygous, yielding a total of 254,299 and 1,316,046 SNPs with heterozygosis rates of 82.20% and 69.14%, respectively. A total of 793,759 SNP markers were found, and these were sorted into eight distinct segregation patterns (aa × bb, lm × ll, nn × np, ab × cc, hk × hk, cc × ab, ef × eg, ab × cd). In the F1 populations, the lm × ll, nn × np, and hk × hk patterns could be used to generate a total of 720,072 SNP markers. The female parent *H. undatus* has 4979 bin markers, a 2710.78 cM map length, an average distance of 0.54 cM, and a maximum gap of 24.8 cM. The male parent *H. polyrhizus* has 2336 bin markers, a 1598.62 cM map length, an average distance of 0.68 cM, and a maximum gap of 79.87 cM. The integrated map has 6209 bin markers, a 2226.22 cM map length, an average distance of 0.36 cM, and a maximum gap of 16.95 cM. These findings indicated that a high-density genetic map has been developed for *Hylocereus* species and can be utilized for future studies.

## 14. Conclusions and Future Perspectives

Pitaya cultivation is still challenged with multiple problems, such as pathogens, regulation of flowering, flower and fruit drop, betalain accumulation, and hot and cold stresses. Recent biotechnological techniques and breeding programs have the capability to overcome these challenges. The selection of premium cultivars with big fruits resistant to fungus and bacterial infection will determine the fruit’s future. Pitaya genotype micropropagation has proven to be successful, with the majority of the success so far coming from organogenesis. Being a natural cactus, the culturing of elite material contains widespread microbial contamination in the majority of tissues, thereby needing surface disinfestation and culture conditions. There have been no known attempts at genetic transformation to tackle certain production issues in prevalent cultivars without changing the key horticultural features. To improve pitaya breeding and germplasm management, diversity analysis and marker-assisted selection will be very helpful. To sum up, it is clear that there are a number of restrictions put on pitaya fruit features and that new methods and instruments must be investigated for pitaya breeding. Horticulturally valuable features, such as superior nutritional qualities, regulation of floral buds, greater resistance to pests and diseases, and diverse ornamental traits, can be induced using techniques like embryo rescue, polyploidy induction, and increasing the usage of mutagenesis or genetic transformation. Pitaya is an exotic fruit on the market, and there is solid evidence that it can be helpful in the fight against cancer. However, there have been legal challenges to these claims. Many studies have been published on the positive effects of pitaya on health and nutrition, but the authors hope to see more research published on the use of biotechnology to further advance this fruit.

## Figures and Tables

**Table 1:** Nutritional value per 100 g FW of different species of pitaya (*Hylocereous* spp.).

Nutritional Compositions	*H. undatus*	*H. polyrhizus*	*H. megalanthus*
Total phenolic content	28.65 mg GAE	24.22 mg GAE	22.90 mg GAE
Carbohydrates	6.26 g	5.97 g	13.07 g
Dietary fiber	0.83 g	1.01 g	1.27 g
Total sugar	6.06 g	5.60 g	5.93 g
Protein	0.94 g	0.89 g	0.40 g
Fat	0.57 g	0.57 g	0.10 g
Iron	0.87 mg	0.78 mg	21.07 mg
Zinc	0.34 mg	0.29 mg	4.35 mg
Sodium	4.50 mg	14.30 mg	1.43 mg
Niacin	0.43 mg	2.80 mg	0.20 mg
Potassium	193.0 mg	158.29 mg	98.41 mg
Phosphorus	29.9 mg	29.2 mg	18.0 mg
Calcium	45.7 mg	31.2 mg	11.7 mg
Magnesium	45.9 mg	33.2 mg	16.1 mg
Glucose	1.58 g	1.33 g	0.99 g
Fructose	2.15 g	2.0 g	3.25 g
Sucrose	2.12 mg	2.54 mg	1.69 g
Sorbitol	2.61 mg	4.52 mg	NA
Vitamin C	5.64 mg	3.40 mg	11.34 mg
Vitamin D2	0.69 μg	0.58 μg	NA
Vitamin E	100.0 μg	140.0 μg	NA
Vitamin K1	30.05 μg	9.40 μg	NA
Refs.	[24,25,26,30,31]	[24,31,32]	[28,33]

Note: The nutritional composition between different species, geographical locations, and methods of determination may differ.

**Table 2:** Summary of the biological activities of pitaya.

Species	Sections	Biological Activity	Refs.
*H. polyrhizus*	Peel	Antioxidant	[42]
*H. undatus*, *H. polyrhizus*	Peel, pulp	Antioxidant	[24]
*H. polyrhizus*	Pulp	Antioxidant	[43]
*H. undatus*	Pulp	Antioxidant	[44]
*H. undatus*	Peel	Antioxidant	[45]
*H. undatus*	Pulp	Antioxidant	[46]
*H. polyrhizus*	Flesh, peel	Antimicrobial, antioxidant	[47]
*H. polyrhizus*	Flesh, peel	Antioxidant, antiproliferative	[48]
*H. undatus*, *H. polyrhizus*	Seed	Antioxidant	[49]
*H. polyrhizus*	Pulp	Antioxidant	[50]
*H. undatus*, *H. polyrhizus*	Peel	Anticancer	[51]
*H. undatus*, *H. polyrhizus*	Peel	Antioxidant, antibacterial against *Bacillus cereus*, *Listeria monocytogenes*, *Staphylococcus aureus*, *Salmonella typhimurium*, *Enterococcus faecalis*, *Escherichia coli*, *Klebsiella pneumonia*, *Yersinia enterocolitica*, and *Campylobacter jejuni*	[52]
*H. polyrhizus*	Pulp	Antioxidant, antibacterial against *S*. *aureus*, *Pseudomonas aeruginosa*, *Candida albicans*, *Aspergillus niger*, *Fusarium oxysporum*	[53]
*H. polyrhizus*	Pulp	Antioxidant, hypocholesterolemic	[54]
*H. polyrhizus*	Pulp	Antioxidant, hypertriglyceridemia, atherosclerosis, insulin resistance	[55]
*H. undatus*, *H. polyrhizus*	Flesh	Antidiabetic, prebiotic	[56]
*H. undatus*	Pulp	Antioxidant, antidiabetic, antilipase activities	[57]
*H. undatus*	Flowers, stems, pulp, peel,	Wound-healing	[58]
*H. polyrhizus*	Peel	Antioxidant, antimicrobial against *E*. *coli*, *Bacillus subtilis*, *S*. *aureus*, *A*. *niger*, *C*. *albicans*	[59]
*H. polyrhizus*	Stem, flower, peel	Antioxidant, wound-healing	[60]
*H. Costaricensis*	Juice	Antianemia, anti-inflammatory	[61]
*H. undatus*	Cladodes	Micro-vascular protective	[62]
*H. polyrhizus*	Pulp	Hepatoprotective	[63]
*H. polyrhizus*	Pulp	Antihyperlipidemic, hepatoprotective, antidiabetic, cardiovascular	[64]
*H. undatus*, *H. polyrhizus*	Flesh, peel	Prebiotic	[65]

**Table 3:** Comparison of pitaya fruit, stem characteristics, and chromosome numbers of the three major *Hylocereus* species cultivated commercially.

Characteristics	*H. undatus*	*H. polyrhizus*/ *H. monacanthus*	*H. megalanthus*
Flower shape	funnel-shape without thorns	funnel-shaped without thorns	goblet-shaped with thorns
Thorns No.	1–4	1–4	≥4
Stem rib No.	triangle	triangle	triangle
Fruit shape	oblong with long scales	round with long scales	oblong without scales
Peel color	red/yellow	red	yellow
Pulp color	white	red/pink/double color	white
Chromosome No.	22	22	44
Refs.	[92,94]	[92,94]	[92,94]

**Table 4:** Summary of the molecular markers of pitaya.

Species	Population Size	Type of Marker	No. of Markers	Refs.
*H. undatus*, *H. ocamponis*, *H. costaricensis*, *H. purpusii*, *H. polyrhizus*, *S. megalanthus*, *S. grandifloras*, *S. coniflorus*, *S. atropilosus*, *S. rubineus*, *S. macdonaldiae*, *S. wercklei*, *S. innesii Kimnach*, *S. murrillii*	34	RAPD	173	[211]
*H. undatus*	16	RAPD	111	[212]
*H. undatus*	13	RAPD	162	[213]
*Hylocereus*	15	RAPD	43	[214]
*H. undatus*	50	RAPD	15	[215]
*H. undatus*, *H. costariscensis*, *H. megalanthus*	4	ISSR	16	[208]
*H. polyrhizus*, *H. undatus*	50	ISSR	111	[210]
*S. megalanthus*	76	ISSR	8	[216]
*H. undatus*	9	ISSR	13	[217]
*H. undatus*, *H. megalanthus*	32	SSR	16	[218]
*Hylocereus*	46	SSR	18	[219]
*H. monacanthus*, *H. megalanthus*	49	SSR	23	[204]
*H. guatemalensis*, *H. undatus*, *H. megalanthus*, *H. polyrhizus*/*H. costaricensis*, *H. ocamponis*	230	AFLP	51	[220]
*H. monacanthus*, *H. undatus*, *H. megalanthus*	59	AFLP	192	[91]
*H. undatus × H. monacanthus*	198	SNP	6434	[221]
*H. undatus × H. polyrhizus*	203	SNP	6209	[222]

## Data Availability

Not applicable.
